# Physiological and kinematic effects of a soft exosuit on arm movements

**DOI:** 10.1186/s12984-019-0495-y

**Published:** 2019-02-22

**Authors:** Michele Xiloyannis, Domenico Chiaradia, Antonio Frisoli, Lorenzo Masia

**Affiliations:** 1 Nanyang Technological University, Robotics Research Center, School of Mechanical & Aerospace Engineering, Singapore, 639798 Singapore; 2 Nanyang Technological University, Interdisciplinary Graduate School, Singapore, 639798 Singapore; 3 Scuola Superiore Sant’Anna, TeCIP Institute, PERCRO Laboratory, Pisa, Italy; 4 Institut für Technische Informatik (ZITI), Faculty of Physics and Astronomy, Heidelberg Universit, Heidelberg, Germany

**Keywords:** Soft exosuit, Assistive wearable robot, Human-robot interaction, Kinematics, Muscular fatigue, Electromyography

## Abstract

**Background:**

Soft wearable robots (exosuits), being lightweight, ergonomic and low power-demanding, are attractive for a variety of applications, ranging from strength augmentation in industrial scenarios, to medical assistance for people with motor impairments. Understanding how these devices affect the physiology and mechanics of human movements is fundamental for quantifying their benefits and drawbacks, assessing their suitability for different applications and guiding a continuous design refinement.

**Methods:**

We present a novel wearable exosuit for assistance/augmentation of the elbow and introduce a controller that compensates for gravitational forces acting on the limb while allowing the suit to cooperatively move with its wearer. Eight healthy subjects wore the exosuit and performed elbow movements in two conditions: with assistance from the device (powered) and without assistance (unpowered). The test included a dynamic task, to evaluate the impact of the assistance on the kinematics and dynamics of human movement, and an isometric task, to assess its influence on the onset of muscular fatigue.

**Results:**

Powered movements showed a low but significant degradation in accuracy and smoothness when compared to the unpowered ones. The degradation in kinematics was accompanied by an average reduction of 59.20±5.58*%* (mean ± standard error) of the biological torque and 64.8±7.66*%* drop in muscular effort when the exosuit assisted its wearer. Furthermore, an analysis of the electromyographic signals of the biceps brachii during the isometric task revealed that the exosuit delays the onset of muscular fatigue.

**Conclusions:**

The study examined the effects of an exosuit on the characteristics of human movements. The suit supports most of the power needed to move and reduces the effort that the subject needs to exert to counteract gravity in a static posture, delaying the onset of muscular fatigue. We interpret the decline in kinematic performance as a technical limitation of the current device. This work suggests that a powered exosuit can be a good candidate for industrial and clinical applications, where task efficiency and hardware transparency are paramount.

**Electronic supplementary material:**

The online version of this article (10.1186/s12984-019-0495-y) contains supplementary material, which is available to authorized users.

## Background

In the never-ending quest to push the boundaries of their motor performance, humans have designed a wealth of wearable robotic devices. In one of the earliest recorded attempts to do so, in 1967, Mosher aspired to create a symbiotic unit that would have the “*...alacrity of man’s information and control system coupled with the machine’s power and ruggedness*” [1]. His design of the *Hardiman*, although visionary, ran into fundamental technological limitations.

Advances in materials science, electronics and energy storage have since enabled an exponential growth of the field, with state-of-the-art exoskeletons arguably accomplishing Mosher’s vision [2]. Wearable robotic technology has been successful in augmenting human strength during locomotion [3], reducing the metabolic cost of human walking [4, 5], restoring ambulatory capabilities to paraplegic patients [6], assisting in rehabilitating stroke patients [7–9], harvesting energy from human movements [10] and helping to study fundamental principles underlying human motor control [11, 12].

These feats were achieved with machines made of rigid links of metal and capable of accurately and precisely delivering high forces to their wearer. While this is undeniably an advantage, it comes at a cost: 1) a significant inertia, which affects both the kinematics of human movement and the power requirements of the device; 2) the need for the joints of the robot to be aligned with the biological joints [13], resulting in increased mechanical complexity and size [14]; 3) a strong cosmetic impact, shown to be linked with psychological health and well-being [15].

The recent introduction of soft materials to transmit forces and torques to the human body [16] has allowed to design wearable robotic devices on the other side of the spectrum: lightweight, low-profile and compliant machines that sacrifice accuracy and magnitude of assistance for the sake of portability and svelteness.

Soft exoskeletons, or exosuits, are clothing-like devices made of fabric or elastomers that wrap around a person’s limb and work in parallel with his/her muscles [17, 18]. Characteristic of exosuits is that they rely on the structural integrity of the human body to transfer reaction forces between body segments, rather than having their own frame, thus acting more like external muscles than an external skeleton. Their intrinsic compliance removes the need for alignment with the joints and their low-profile allows to wear them underneath everyday clothing.

Exosuits actively transmit power to the human body either using cables, moved by electric motors, or soft pneumatic actuators, embedded in the garment. The latter paradigm was probably among the first to be proposed [19] and has been explored to assist stroke patients during walking [20], to increase shoulder mobility in subjects with neuromuscular conditions [21], to help elbow movements [22] and for rehabilitation purposes to train and aid grasping [23–25].

Cable-driven exosuits, instead, include a DC motor that transmits power to the suit using Bowden cables. This flexible transmission allows to locate the actuation stage where its additional weight has the least metabolic impact on its wearer. Using this paradigm to provide assistance to the lower limbs has resulted in unprecedented levels of walking economy in healthy subjects [26] and improved symmetry and efficiency of mobility in stroke patients [27]. Similar principles were used to provide active support to hip and knee extension, reducing activation of the gluteus maximus in sit-to-stand and stand-to-sit transitions [28].

Cable-driven exosuits seem to work particularly well for lower-limbs movements, where small bursts of well-timed assistance can have a big impact on the dynamics and metabolic cost of locomotion [29]. Yet, Park et al. have shown that they have the potential for assisting the upper-limbs in quasi-static movements too: using a tendon-driving mechanism, a textile interface and an elastic component they found a significant reduction in the activity of the deltoid muscle when supporting the weight of the arm [30].

Similar results were reported by Chiaradia et al., where a soft exosuit for the elbow was shown to reduce the activation of the biceps brachii muscle in dynamic movements [31], and by Khanh et al., where the same device was used to improve the range of motion of a patient suffering from bilateral brachial plexus injury [32].

While there is extensive work on the analysis of the effects of wearing a soft exosuit on the kinematics, energetics and muscular activation during walking [33], the authors are unaware of comparable studies on movements of the upper limbs, whose variety of volitional motions is fundamentally different from the rhythmic nature of walking.

Understanding how these devices affect the physiology and mechanics of human movements is fundamental for quantifying their benefits and drawbacks, assessing their suitability for different applications and guiding a continuous data-driven design refinement.

In this study we investigate the kinematic and physiological effects of wearing a cable-driven exosuit to support elbow movements. We hypothesize that the low inertia and soft nature of the exosuit will allow it to work in parallel with the user’s muscles, delaying the onset of fatigue while having little to no impact on movement kinematics.

We propose a variation of the design and controller presented in [32, 34] and introduce a controller that both detects the wearer’s intention, allowing the suit to quickly shadow the user’s movements, and compensates for gravitational forces acting on the limb, thus reducing the muscular effort required for holding a static posture. We collect kinematic, dynamic and myoelectric signals from subjects wearing the device, finding that the exosuit affects motion smoothness, significantly reduces muscular effort and delays the onset of fatigue. The analysis offers interesting insights on the viability of using this technology for human augmentation/assistance and medical purposes.

## Methods

### Exosuit design

An exosuit is a device consisting of a frame made of soft material that wraps around the human body and transmits forces to its wearer’s skeletal structure. In a cable-driven exosuit, artificial tendons are routed along a targeted joint and attached to anchor points on both of its sides. When the tendons are tensioned they deliver an assistive moment to the joint.

**Fig. 1:**
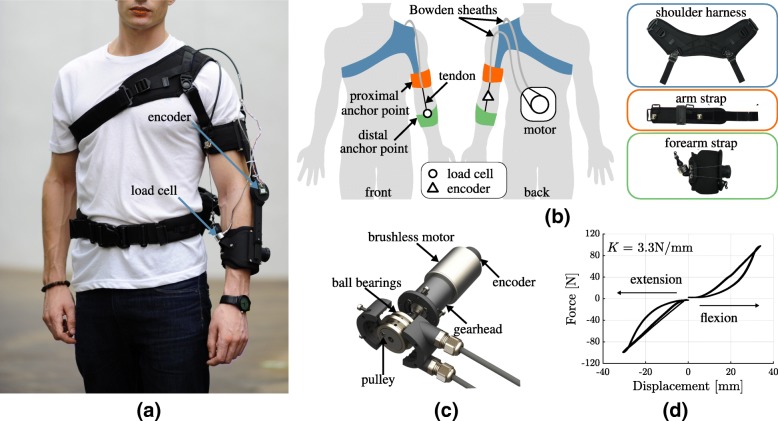
Design and actuation of the soft exosuit for the elbow. **a-b** The exosuit comprises three straps that wrap around the shoulder, arm and forearm, highlighted in blue, orange and green, respectively. The last two act as anchor points: the Bowden cables’ outer sheath is attached to the arm strap and the inner tendons to the forearm strap. A load cell and an encoder sense the interaction force and the elbow position. **c** The actuation stage comprises a brushless motor, equipped with a gearhead and encoder, that drives a spool around which the suit’s tendons are wrapped. **d** Stiffness of the exosuit. The Bowden cables and the fabric introduce compliance in the transmission, this series-elasticity can be exploited to achieve a safe and robust interaction-force control.Photography by ⒸStefano Mazzoni

The exosuit for assistance of the elbow joint presented in this paper (shown in Fig. 1a, b) follows exactly this principle. It comprises of three fabric straps: one around the forearm (distal anchor point), one around the arm (proximal anchor point) and a shoulder harness, connected to the arm strap via adjustable webbing bands. Buckles, velcro straps and a Boa lacing system allow to tighten the suit.

A pair of Bowden cables transmits power from an actuation unit to the anchor points. The Bowden cables sheaths (Shimano SLR, ⊘ 5mm) are attached to the arm strap, while their inner tendons (Dupont, Black Kevlar Fiber, 136kg max load) to the forearm strap. When either of the two tendons is shortened, it pulls together the two anchor points, applying a flexing or extending moment on the elbow.

The shoulder harness is connected via inextensible webbing bands to the arm strap, covers the shoulder and encircles the chest; its purpose is to prevent the arm strap from migrating towards the center of the joint by relying on reaction forces from the shoulder and ribcage. The same is achieved for the forearm strap by tightening it with a boa lacing system, the conic shape of the forearm contributes to prevent slippage.

The proximal and chest straps were made by modifying a commercially available passive orthosis (Master-03, Reh4mat). Their substrate is made of a 3-layered fabric: an external layer used to attach hard components (buckles and webbing strips), an intermediate ethylene-vinyl acetate (EVA) foam to avoid peaks of pressure and an internal 3D polyamid structure to provide air permeability. The distal anchor point consists of a flexible plastic sheet, lined with ballistic nylon and covered by a 3mm-thick layer of polyethylene (PE) sponge at the interface with the skin. A load cell (Futek, LCM300), secured on the distal anchor point, measures the tension in the flexing tendon and an absolute encoder (AMS, AS5047P, 1000 pulses/rev), mounted on a 3D-printed joint (Shapeways, versatile plastic) between the arm and forearm straps, senses the angular position of the elbow. The plastic joint, featuring a rotational Degree of Freedom (DoF) at the elbow and a translational DoF at the distal anchor point, bears no loads and does not transmit torque. It thus serves the purpose of a goniometer without altering the fundamental characteristic of an exosuit: to rely on the structural integrity of the human joint to transmit forces between body segments.

### Actuator design

The unit actuating the Bowden cables is shown in Fig. 1c. It consists of a brushless electric motor (Maxon, EC-i 40, 70W) in series with a planetary gearhead (Maxon, GP 32, 55:1), capable of delivering up to 8.5Nm of continuous torque at the elbow joint (sufficient for activities of daily living [35]), and whose angular position is monitored by an incremental encoder (Scancon, 2RMHF, 5000 pulses/rev).

The gearhead’s output shaft drives a pulley around which the two tendons are wrapped in opposite directions, in an antagonistic fashion. The pulley is enclosed in a plastic casing; three ball bearings between the pulley and the plastic prevent the tendons from derailing when they are slack.

The suit’s components and the Bowden cables introduce a fair amount of elasticity in the transmission of power between the motor and the user. Figure 1d shows a characterisation of the exosuit’s stiffness on a rigid mannequin using the methodology described in [36], consisting in commanding the motor to apply a force of 100N on the flexing/extending tendon and measuring its displacement. The device has a quasi-linear behaviour in the loading phase and a non-linear behaviour when unloaded, in both flexion and extension. A least-square linear approximation of its stiffness yields a value of 3.3 N/mm.

While on one hand this series-elasticity is an undesirable property because it lowers transmission efficiency and position-control bandwidth, on the other it introduces well-known advantages in terms of safety and force control accuracy and stability [37].

### Controller

The control algorithm is designed to have the dual purpose of 1) providing assistance by compensating for gravitational forces and 2) not obstructing natural movements, i.e. allow the exosuit to move in concert with its wearer with minimal interaction force between the two.

The first objective requires the ability to track a position-dependent force profile equal and opposite to the gravitational force acting on the forearm. Indirect force controllers, encompassing impedance and admittance architectures, are a common choice to safely interact with human beings [38]. This is because imposing a relation between force and velocity, unlike direct force paradigms, allows to control the power transfer between the device and its user [39].

The second objective requires transparency of the suit to the user’s movements, in other words backdrivability. This cannot be achieved mechanically because the high reduction ratio of the motor’s gearhead increases the reflected motor impedance and the Bowden cables make the transmission inefficient. We need to achieve backdrivability by control.

**Fig. 2:**
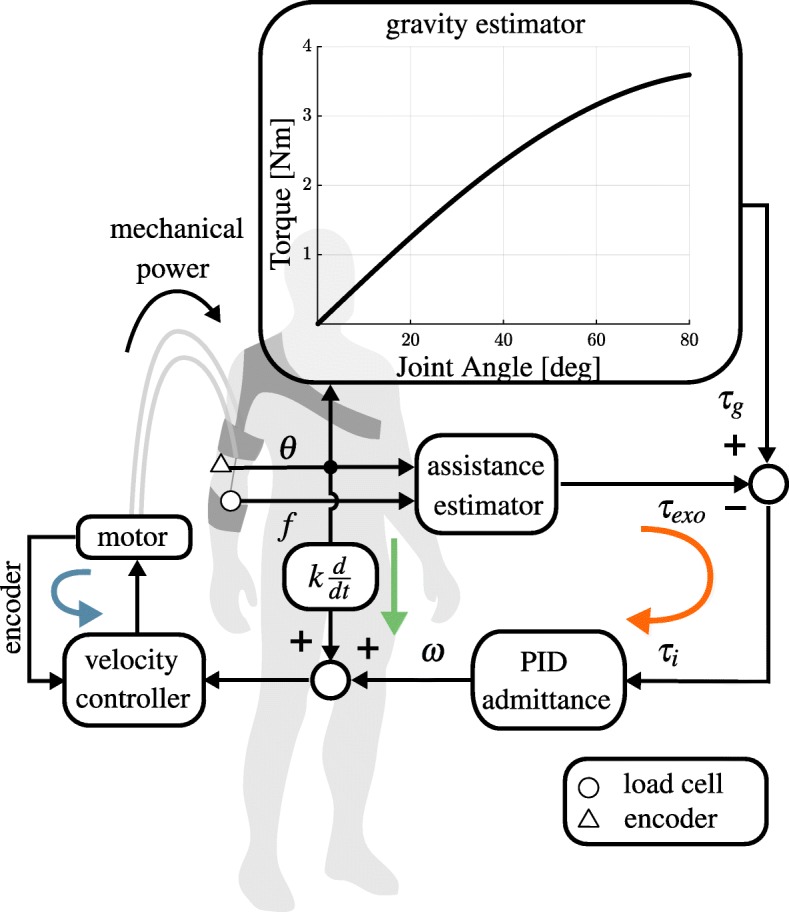
Admittance controller for transparency and gravity compensation. An outer torque loop (orange) tracks a reference profile equal and opposite to gravity, computing a motion reference as an interaction torque is sensed, according to the admittance specified by a PID controller. The inner velocity loop (light blue) is tuned to be as stiff as possible, to reject force disturbances like stiction and backlash. The green arrow indicates a positive feedback path, introduced to improve transparency

The proposed controller is shown in Fig. 2. It comprises an outer torque loop and an inner velocity loop. The former is responsible for tracking the position-dependent torque profile at the elbow, equal and opposite to gravity. In practice, it computes a motion reference as an interaction torque is sensed, thus creating virtual backdrivability.

The goal of the inner velocity loop is to be as fast as possible, following the velocity reference from the outer loop and stably rejecting nonlinearities in the transmission. Differently from the classical admittance implementation, this inner velocity loop is closed at the motor level instead of at the joint level. This approach, known as collocated admittance control [38], has been shown to robustly deal with force disturbances such as stiction and backlash [40], abundant in the soft exosuit.

The torque acting on the elbow joint as a result of gravity is estimated using a simple single-joint model and assuming that the arm is adducted on the side of the trunk:

1 $$ \tau_{g}={mgl}_{c}\sin\theta,   $$

with *m* being the combined mass of the forearm and hand, *l*_*c*_ the distance of the center of gravity of the forearm and hand from the center of rotation of the elbow joint, *g* the acceleration of gravity and *θ* the elbow angle, assumed to be zero in the fully-extended configuration.

The assistive torque is estimated by multiplying the tension measured by the load cell, *f*, by its moment arm *P*(*θ*) (refer to Additional file 1 for a full formulation of the control laws):

2 $$ \tau_{exo}=P(\theta)f.   $$

The difference between *τ*_*g*_ and *τ*_*exo*_, i.e. the interaction force, *τ*_*i*_, between the suit and its wearer, is converted to a reference velocity *ω* for the motor by a specified admittance. Being one of our requirements that of transparency, *τ*_*i*_ must be set to zero. The admittance can assume the form of a PID controller [41]:

3 $$ Y(s) = \frac{\omega}{\tau_{i}} = P + \frac{I}{s} + Ds,   $$

with the P, I and D constants governing the characteristics of the relation between the interaction force and the exosuit’s kinematics. The PID parameters were initially set using the tuning rules described in [41] from the human elbow impedance parameters identified in [42]. A heuristic fine-tuning for each subject was performed in a familiarisation phase prior testing the device.

An additional positive feedback term, proportional to the speed of the elbow joint, increases the sensitivity of the device to its wearer’s movements. As elegantly discussed in [43], this comes at the expense of a loss in robustness, so extra care needs to be taken when tuning the outer admittance loop.

The inner velocity loop ran on a motor controller (Maxon, EPOS2 50/5) and the outer loop on a real-time data acquisition board (Quanser, QPIDe), both at a sampling rate of 1 kHz.

### Experiment

The aim of the evaluation procedure was to assess the effect of the exosuit on human kinematics and biomechanics. To do so, we compared smoothness and accuracy of movement, biological torque and muscular activation patterns of healthy subjects performing controlled motions of the elbow, with and without assistance from the suit.

The testing was done on 8 male subjects (average age 29.2±1.4) presenting no evidence or known history of skeletal or neurological diseases, and exhibiting intact joint range of motion and muscle strength. At the beginning of each experimental session the participants were informed of the procedure and they signed an informed consent. The procedures, in agreement with the Declaration of Helsinki, were approved by the Institutional Review Board at Nanyang Technological University.

**Fig. 3:**
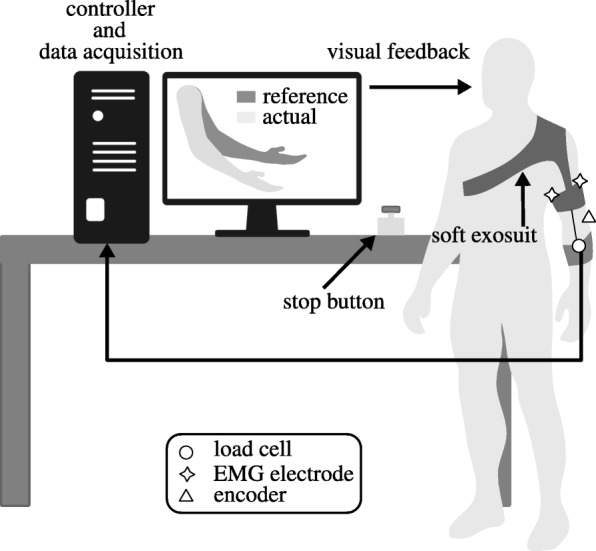
Experimental setup. Subjects were asked to follow a reference trajectory displayed on a screen in the form of a moving elbow, the position of their own arm was superimposed to provide visual feedback. This was done in both the powered and unpowered conditions, while monitoring the elbow angle, the interaction force (only powered) and EMG activity of two antagonistic muscles driving the joint

The experimental setup is shown in Fig. 3. Participants, wearing the exosuit on their left arm, had to follow a reference movement performed by a dummy character on a screen. The position of their own elbow was displayed as a superimposed translucent replica of the reference one to provide visual feedback. To ensure that they were moving at the desired velocity, participants were asked to match the movement of the character on the screen as accurately as possible.

This was done by each subject in two conditions: with and without assistance from the device, we shall refer to these as *powered* and *unpowered* conditions, respectively. In the latter case the exosuit’s tendons were unhooked from the distal anchor point and the motor’s power source was turned off. The sequence of the two conditions was randomly assigned to each participant to mitigate potential order effects.

The reference motion consisted of series of Minimum Jerk Trajectories (MJT), known to correspond well to the movements of healthy subjects [44], at varying peak velocities, chosen to be fractions of the average elbow speed in activities of daily living (ADLs), i.e. 126 deg/s [45]. The evaluation comprised three sessions: a familiarisation phase, a dynamic and an isometric task.

#### Familiarisation

The familiarisation was performed with assistance from the exosuit so that the participant could get accustomed to using the device and we could fine-tune the gains of the PID admittance controller. The participant was asked for his weight and height, used to evaluate the geometrical and physiological parameters used in Eqs. 1-2 and 4, from anthropometric tables [46].

**Fig. 4:**
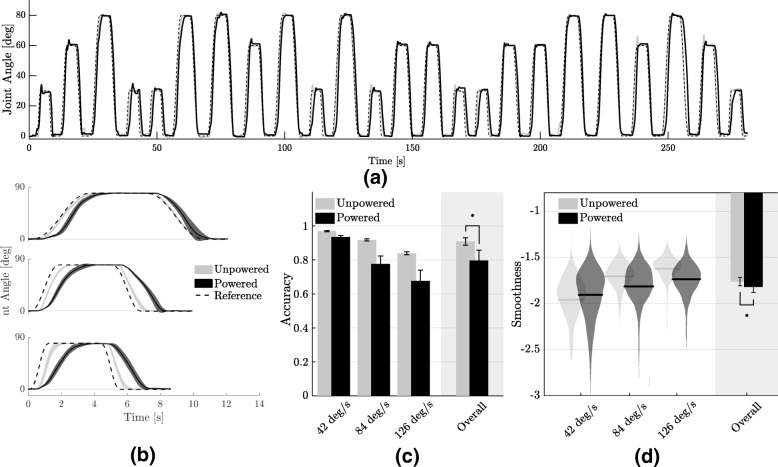
Effect on joint kinematics. **a** Typical sequence of flexion/extension movements performed by a participant in a session with a peak velocity of 42 deg/s. Shaded areas indicate the standard deviation around the mean trajectory. **b** Trajectories for the unpowered and powered conditions, averaged over repetitions, for one subject and at the three tested velocities (from top to bottom: 42, 84 and 126 deg/s); as the velocity increases, the accuracy of the powered condition decreases. **c** Average accuracy, measured through the coefficient of determination, r^2^, between the reference and measured trajectory of the elbow in the unpowered and powered condition. The overall mean, averaged over subjects and velocities, indicate that the assistance from the exosuit significantly (p = 8×10^−5^) reduces a subject’s capacity to follow a reference motion. **d** A similar trend was found for the smoothness of movement, measured with the SPARC index [50]. Assistance from the exosuit significantly reduces movement smoothness (−3.4*%*, p = 2×10^−13^). Error bars show the standard error of the mean

The reference motion consisted in a series of MJTs between 0 deg and 30 deg, 60 deg or 80 deg, each amplitude repeated 8 times in a random order, for a total of 24 movements. A typical reference signal is shown in Fig. 4a. The peak velocity of movement was chosen to be 50% of the average elbow speed in ADLs. No physiological data was recorded.

#### Dynamic task

The dynamic task was used to assess the effectiveness of the exosuit in both shadowing the wearer’s movements and compensating for gravitational forces.

Subjects were asked to hold a mass in their hand and follow the reference trajectory displayed on the screen; this was done both with and without assistance from the exosuit and for three different velocities of movement, for a total of 6 sessions. All sessions of the same condition were performed on the same day, with a 30 min break between them. Powered and unpowered bouts were conducted on separate days to avoid fatigue. The mass consisted in a 1 kg plate, used to increase muscular activation in both conditions and enhance the signal-to-noise ratio of the data collected using surface electromyography.

The reference MJT motion was the same as the one for the familiarisation phase (an example of which can be seen in Fig. 4a) but performed in three different sessions at three different peak velocities: 42 deg/s, 84 deg/s and 126 deg/s, corresponding to 33%, 67% and 100% of the average elbow speed in ADLs.

We recorded the angular position of the elbow, the tension on the exosuit’s flexing tendon and the electromyography (EMG) of the biceps brachii and the long head of the triceps brachii, responsible for flexing and extending the elbow, respectively. The skin was cleaned and the electrodes (Delsys Trigno IM) were placed according to the SENIAM standards [47]. At the beginning of each session we performed a manual test for maximum voluntary contraction (MVC), subsequently used to normalise the muscular activity, allowing comparison across subjects. The test was repeated two times per muscle, with a break in-between to avoid fatigue. All data was acquired at a sampling frequency of 1 kHz through a Quanser QPIDe acquisition board.

#### Isometric task

The goal of the isometric task was to assess the impact of the exosuit on muscle fatigue.

While holding a load, subjects were asked to repeatedly maintain the elbow in a fixed position. The load was chosen to be a mass equivalent to 3% of the participant’s body weight, corresponding to approximately 15% of his MVC, held at 90 deg for three fatiguing repetitions of 40 s each, separated by 20 s of rest. This was done both for the powered and unpowered condition, in a randomized order and on different days. Although fatiguing protocols often involve higher loads and isometric contractions until voluntary exhaustion [48], our suit was not designed to transmit heavy weights to the human body. This combination of magnitude and timing of exercise was chosen as a reasonable compromise between intensity and comfort.

We recorded EMG of the biceps brachii and the long head of the triceps brachii using the same procedure adopted for the dynamic task. One subject was dropped out of the fatigue evaluation due to incorrect placement of the electrodes.

### Data Analysis

Raw data from the suit’s absolute encoder and load cell was low-pass filtered (second order Butterworth filter, 10 Hz cut-off frequency) and segmented to isolate the 24 movements comprising each session.

The accuracy of movement was quantified by evaluating the coefficient of determination (r^2^) between the measured and reference trajectory. Time delays between the reference and measured trajectories were estimated by finding the time lag corresponding to a peak in the cross-correlation between the two signals.

Smooth movements are a characteristic feature of healthy, efficient and well-trained motor behaviour [49] and an external assistive device should not make them less so. To quantify kinematic smoothness, we used the SPectral ARC length (SPARC) index proposed in [50]. This required an additional event-based segmentation to isolate epochs where subjects were actually moving from those of static holding, which we did using a lower threshold on the absolute velocity of 2.5 deg/s. The SPARC index was estimated on the norm of the elbow’s speed.

The measured force on the flexing tendon was mapped to a torque on the joint using Eq. 2, this was used as an estimate of the assistive moment delivered by the exosuit, *τ*_*exo*_. The total torque required to perform the movement was derived from the inverse dynamics of the human elbow, represented as a simple pendulum using a second order model of the form:

4 $$ I\ddot{\theta}+B\dot{\theta}+\tau_{g}= \tau,   $$

with *I* being the moment of inertia of the forearm and hand, B takes into account the viscosity of the elbow joint (we used a value of 0.2 Nms/rad according to the values reported in [42]) and *τ*_*g*_ is the gravity-dependent torque, presented in Eq. 1. The norm of the difference between the total and assistive torque, *τ*_*bio*_=*τ*−*τ*_*exo*_, was used to estimate the remaining biological torque exerted by the subject to perform the movement or hold the position. The absolute value of the biological torque |*τ*_*bio*_| was used as a cost index (the higher the worse) of the performance of the device.

The output EMG signal of the Delsys Trigno system (pre-conditioned with a band-pass Butterworth filter between 20 Hz and 450 Hz) was processed to extract its linear average envelope using the procedure suggested in [51]: this included noise filtering, rectification, smoothening using a moving-average filter (0.2 s window) and normalisation by the MVC. The root mean square (RMS) of the processed EMG signal was used as index of the level of activation of a muscle.

Finally, the EMG data gathered from the isometric task was used to evaluate the effect of the exosuit on the onset of fatigue. Myoelectric manifestations of muscle fatigue appear both in the time and frequency domain as an increase in the EMG amplitude or as a shift towards lower frequencies of the signal’s power spectral density function [52]. We used the median frequency (MNF) of the EMG’s power spectrum and the average rectified value (ARV) of its amplitude as indexes of fatigue, evaluated on epochs of 3 s during the last isometric repetition. The rate of change of these values during the 40 s of isometric contraction were used to quantify fatigue. We calculated their slope by fitting a first order model with a least square method: a steeper positive slope for the ARV and a steeper negative one for the MNF indicate a faster onset of fatigue.

### Statistical analysis

We checked that the metrics were normally distributed using a Shapiro-Wilk test with a significance level of *α*= 0.05. All metrics were normally distributed except for the elbow’s smoothness (SPARC index) and coefficient of determination (r^2^) between the reference and measured trajectories.

Non normally-distributed metrics were evaluated by a non-parametric Wilcoxon signed-rank test between the powered and unpowered conditions, our null hypothesis being that both samples came from distributions with equal mean. Normally-distributed metrics were statistically compared with a paired t-test (*α*= 0.05) between the powered and unpowered conditions. Outliers were removed before any further analysis using a Thompson Tau test.

Reported values and measurements from here onwards, in both graphs and text, are presented as mean ± standard error of the mean (SEM).

## Results

### Wearing the exosuit reduces movement accuracy and smoothness

Figure 4 shows the effect of the exosuit on the trajectories of the elbow. As shown in Fig. 4b, as the velocity of movement increased, the tracking accuracy of the powered condition worsened when compared to the unpowered one. The average accuracy, measured by the coefficient of determination between the measured and reference trajectories, for the powered and unpowered conditions were 0.91±0.02 and 0.80±0.06, respectively. A Wilcoxon signed-rank test between the two confirmed that wearing the exosuit significantly reduces the ability to track a reference trajectory (*p* = 8×10^−5^).

**Fig. 5:**
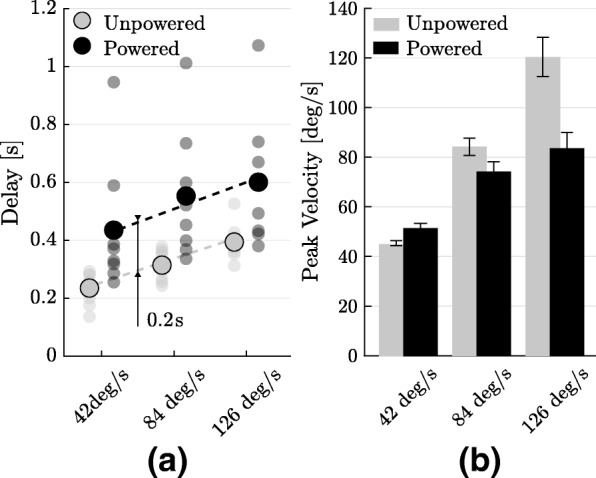
Effect of the exosuit on delay and peak velocity of movement. **a** Wearing the exosuit introduced a time lag between the reference trajectory and the wearer’s movement. The average delay, over subjects, is 200 ms higher than the one observed in the unpowered condition, independently of the target velocity. **b** Assistance from the exosuit slowed down human movement for velocities higher than 42 deg/s. This is most most probably a corollary of the limited bandwidth of the device (Additional file 1)

This deterioration in tracking accuracy is a consequence of both a delay introduced by the suit in the initiation of movement and its inability to track high velocities. The former effect is shown in Fig. 5.a, highlighting that the suit offset reaction times by approximately 200 ms, independently of movement speed. Figure 5b shows that wearing the suit slowed down human movements. Although this was observed overall, averaging over velocities and subjects, it did not apply to low velocities (42 deg/s), where the opposite was true.

Similarly, the smoothness of movement was affected by the exosuit’s assistance, with the difference in SPARC index [50] between the two conditions increasing for increasing movement velocity. The overall smoothness, averaged over velocities and subjects, was −1.76±0.10 (unpowered) and −1.82±0.14 (powered). The latter being significantly lower than the former (*p* = 2×10^−13^).

### Wearing the exosuit reduces muscular effort

**Fig. 6:**
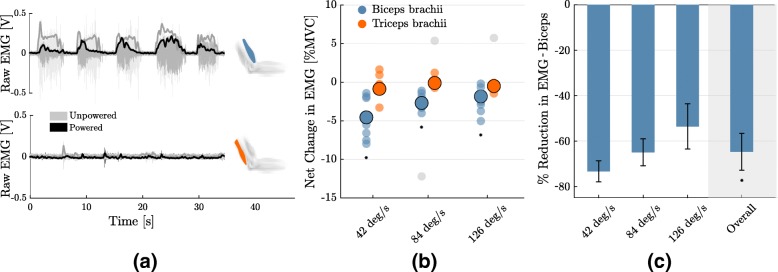
Changes in muscular activation. **a** Raw signal and envelope of the electromyography (EMG) of the biceps brachii and long head of the triceps brachii during five consecutive movements, performed in the powered (black) and unpowered (grey) conditions. **b** Net change (powered - unpowered) of the root mean square of the EMG signal of both evaluated muscles, for the three velocities. Translucent circles are the values for each individual subject, opaque contoured circles indicate the mean over subjects. Circles in grey are outliers, identified through a Thomson tau analysis. Asterisks indicate significant difference from 0. **c** Change in the activity of the biceps brachii, expressed as percentage of its activity in the unpowered condition (net change/unpowered). Assistance from the exosuit singificantly reduces muscular effort (64.8±7.66*%*, p = 5×10^−15^). Error bars show the standard error of the mean

As the suit provided a force against gravity to support the weight of the forearm, it reduced the amount of effort that the flexor muscle needed to exert (*p* = 5×10^−15^). Figure 6a shows a representative case of the activity (raw and its envelope) of the biceps brachii and long head of the triceps brachii during five consecutive movements of the elbow, in both the powered and unpowered conditions.

The net change in the biceps brachii muscular effort (Fig. 6b), evaluated as the difference in the EMG’s RMS between the powered and unpowered cases, was significantly smaller than 0 for all velocities (*p* =1×10^−3^ for 42 deg/s, *p* =1×10^−3^ for 84 deg/s, *p* =8×10^−3^ for 126 deg/s). Such was not the case for the triceps brachii, whose activity’s net change between the two conditions cannot be said to differ from 0.

Figure 6c shows the change in activity of the biceps brachii expressed as percentage of its activity in the unpowered condition. Similarly to what happened to the accuracy and smoothness of movement (Fig. 4), the performance of the suit degraded for higher velocities. Wearing the exosuit resulted in a significant reduction of the biceps muscle effort, averaged over subjects and velocities, of 64.8±7.66*%*(*p* = 5×10^−15^).

### Wearing the exosuit reduces the biological torque required for movement

**Fig. 7:**
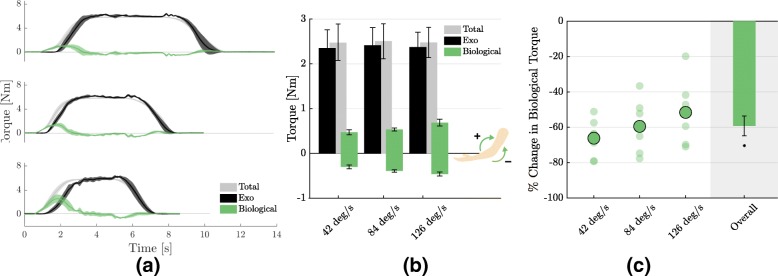
Changes in biological torque. **a** Total, assistive torque provided by the exosuit and biological torque (total-exo) for one subject, averaged over repetitions, for all three velocities of movement (from top to bottom: 42, 84 and 126 deg/s). As the velocity of movement increases, the magnitude of the biological torque increases, mostly around the transient regions. Shaded areas indicate the standard deviation around the mean. **b** Average over subjects of the total, assistive and biological torques. The exosuit compensates for most of the positive (flexing) torque but introduces a negative (extending) component. **c** Change in biological torque, expressed as percentage of the total torque in the unpowered condition (|biological powered | /total unpowered). Translucent circles are the values for each individual subject, opaque contoured circles indicate the mean over subjects. Asterisks indicate significant difference from 0. Wearing the exosuit significantly reduces the magnitude of the torque that the wearer needs to exert to move (−59.20±5.58*%*, *p* = 9×10^−14^). Error bars show the standard error of the mean

Figure 7a shows the total torque required to perform the movement (grey), the one provided by the exosuit (black) and the estimated biological torque (in green), for one subject, averaged over repetitions for the three tested velocities of movement. The exosuit supports large part of the total torque but introduces negative biological moments, especially when initiating the downwards motion.

Figure 7b shows the average over subjects and repetitions of the total, the exosuit’s and the biological torque in the powered condition (in the unpowered condition *τ*_*bio*_=*τ*_*total*_). The figure shows that when the exosuit is assisting the subject, the biological torque is only a fraction of the total one but, as the velocity increases, the wearer needs to exert higher positive and negative torques.

The overall gain, shown in Fig. 7c, was, nevertheless, favorable, with the percentage change of the absolute biological torque (|*τ*_*bio*_|) between the powered and unpowered conditions being significantly lower than 0 for all individual velocities (*p* =3×10^−6^ for 42 deg/s, *p* =4×10^−5^ for 84 deg/s, *p* =3×10^−4^ for 126 deg/s) and overall (−59.20±5.58*%*,*p* = 9×10^−14^).

### Wearing the exosuit delays the onset of fatigue

The isometric contraction task, performed with aid from the exosuit, showed a slower onset of fatigue in the biceps brachii compared to the unpowered condition (*p* = 0.03 for the ARV and *p* = 0.01 for the MNF).

**Fig. 8:**
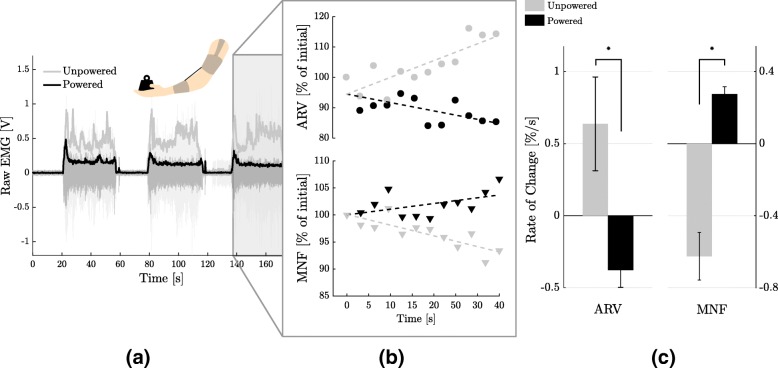
Fatigue analysis. **a** Raw signal and envelope of the electromyography (EMG) of the biceps brachii of one subject, during the isometric task, for both the unpowered and powered condition. **b** Trend of the average rectified value (ARV) and median frequency (MNF) of the EMG signal of one subject, during the last isometric contraction. Indexes are expressed in percentage of their initial value. A steeper positive slope for the ARV and a steeper negative one for the MNF indicate a faster onset of fatigue [52]. **c** The slope of the ARV and MNF, averaged over subjects. Both indexes confirm that wearing the exosuit significantly reduces the onset of fatigue (*p* = 0.03 for the ARV and *p* = 0.01 for the MNF). Error bars show the standard error of the mean

Figure 8a-b show the raw and envelope of the biceps’ EMG signal, and the trend of the average rectified value (ARV) and median frequency of the EMG’s spectrum (MNF) for both the powered and unpowered conditions of one representative subject. Values over the 40 s contraction window are reported in percentage of the initial value, discarding the first 3 s after reaching the target position of the elbow. A steeper positive slope for the ARV and a steeper negative one for the MNF indicate a faster onset of fatigue.

The mean slope and its standard error over subjects are shown, for both metrics and conditions, in Fig. 8c.

## Discussion

Trading limited force output and control accuracy for portability and svelteness, soft wearable robots have the potential to become a ubiquitous part or our daily lives in the near future. Understanding how the assistance of soft exosuits impacts on the biomechanics of human movements is crucial for designing better hardware, developing more effective control paradigms and systematically assess their suitability for being used in daily life.

In this study we have proposed a refined version of the exosuit for the elbow previously described in [34, 53] and introduced a simple controller to provide intuitive assistance to the suit’s wearer by following his/her movements while removing gravitational forces.

Moving with assistance from the powered exosuit lowered the muscular effort by an average of 64.8±7.66*%*. This is probably a direct corollary of the observed significant reduction in biological torque between the powered and unpowered conditions.

These findings are in line with what we detected in a preliminary evaluation of the exosuit on two subjects, described in Chiaradia et al. [31], and show a higher benefit when compared to our very first evaluation of an assistive sleeve, reported in Dinh et al. [34].

The results in [34] (48.5*%* reduction in muscular effort) are only indicatively comparable to the ones presented here. The difference is partly caused by a refinement of the hardware and partly by considering that the control approach presented in Dinh et al. was aimed at assisting impaired subjects and designed to automatically tailor the level of assistance to the ability of movement of its wearer.

**Fig. 9:**
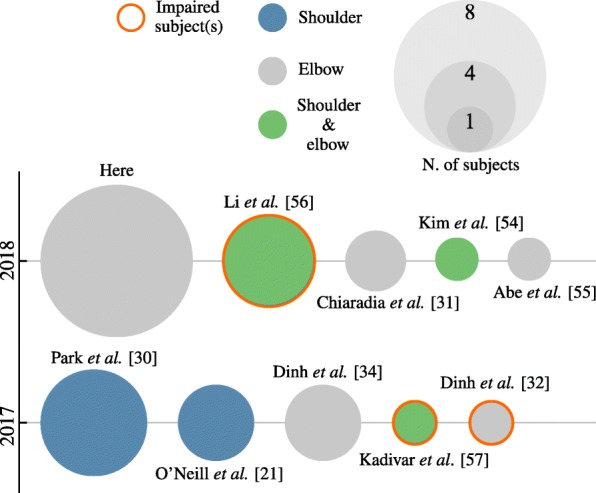
Recent studies that report the effect of soft wearable robots on the biomechanics of shoulder and/or elbow movements

A systematic comparison of our work with existing literature is not yet possible because of the absence of a standard assessment procedure. Figure 9, however, highlights recent works that specifically report biomechanical and muscular effects of wearing a soft exosuit on joints that, like the elbow, are involved in gross lifting tasks. The figure puts emphasis on sample size and population type.

Four of the studies listed in Fig. 9 use the reduction in magnitude of the EMG activity as a performance index. Kim et al. [54] report changes in muscular activation when wearing a cable-driven exosuit assisting shoulder and elbow flexion. During a static task, one subject showed an average of 49.4*%* and 68% reduction in the biceps brachii and the anterior deltoid, respectively.

Similarly, O’Neill et al. [21] present a wearable robot for the shoulder that uses soft textile pneumatic actuators to assist the joint in abduction and horizontal flexion/extension. The device, evaluated on three healthy participants, reduced the activity of the medial deltoid (63.89*%*) and infraspinatus muscles (34.03*%*) when abducting the shoulder and that of the pectoralis major (23.20*%*) and posterior deltoid (70.09*%*) during horizontal flexion/extension.

Abe et al. present a suit made of pressurised muscle textile; they evaluated it on one subject and reported a reduction in the EMG activity of the biceps brachii of 33% and an increase in that of the triceps brachii of 35% [55].

Li and colleagues [56] use a paradigm similar to the one presented here to assist healthy subjects and stroke patients in flexing the shoulder and elbow. They recorded a reduction in the activity of the biceps brachii of a healthy individual of 58.17% and an increase in the range of motion of chronic stroke patients of up to 174%.

Two more studies evaluate the effect of exosuits on the movements of the upper limbs of impaired participants. Dinh et al. [32] use the residual EMG activity of a severe brachial plexus injury patient to initiate a flexing movement of the elbow. Kadivar et al. [57] explore the feasibility of introducing a similar device for the shoulder and elbow in a rehabilitation procedure of a traumatic brain injury patient. None of these two studies, though, report changes in muscular activity.

It is worth highlighting that all the results listed in Fig. 9 were obtained with a procedure fundamentally different from the one used in this study: the admittance controller that we propose allowed the suit to continuously move in concert with its wearer while delivering an assistive torque. The other studies listed here lacked an intention-detection strategy. The robot was triggered to apply a predefined torque or trajectory, regardless of the intention of the wearer: during the evaluation the subject was simply asked to relax.

Moving with the exosuit reduced the biological torque by an average of 59.20±5.58*%*. The device could compensate the forearm’s weight nearly entirely while holding a static position but the wearer still had to make a significant effort at initiation of movement (see the positive peaks of biological torque in Fig. 7a). This limitation was most likely caused by the flexing tendon slacking when the arm was fully extended; assistance was not delivered at initiation of movement until the slack was recovered. A simple solution to this problem could be to pre-tension the tendons. This, however, might negatively impact on comfort, especially during prolonged use.

Moving with the exosuit increased the extending biological torque at the elbow compared to the unpowered condition (+0.39 Nm). Participants needed to slightly push to initiate the downwards motion when the elbow was flexed (see the peaks of negative torque in Fig. 7a). This unwanted interaction torque was caused by the impedance of the controller and can be used as an index of the transparency of the exosuit: increasing the admittance of the controller would reduce this effect but would make the device less stable.

Surprisingly, the increase in negative torque was not accompanied by a significant increase in the activity of the extensor muscle. One plausible explanation could be the subtle change in the activation of the triceps brachii was not sufficient in magnitude to be detected with surface EMG. Further investigations, looking, for example, at the muscular response while holding heavier weights, could help to clarify this point.

During isometric tasks our exosuit delayed the onset of fatigue. This result is most likely a corollary of the observed reduction in biological torque. A similar finding is described in [30], where a cable-driven suit for the shoulder is shown to reduce the fatigue in the anterior and medial deltoid of five healthy subjects. Unfortunately, a quantitative comparison here is not possible because of the different metrics used to assess fatigue in [30].

Moving with assistance from the exosuit significantly reduced the accuracy of movement. This deterioration in accuracy was caused by the powered movements being slower than the unpowered ones. Figure 4b shows a clear delay between the reference and measured trajectory of the elbow in the powered condition, quantified in Fig. 5a. Wearing the exosuit introduced a delay in the reaction time of approximately 200 ms. An analysis of the peak velocities of the elbow between the two conditions (Fig. 5b) confirms these observations: wearing the exosuit reduces the peak velocity of the elbow by an average of 9.4 ±4.4%.

This slowing down of movements is consistent with previous findings investigating the effects of interactions with an exoskeleton on human motion [58], but its underlying cause is not entirely clear. We think that one or a combination of two mechanisms may be at play here: (1) Desmurget et al. [59] have shown that movements constrained by contact with an external body (in this case the exosuit), involve a fundamentally different control strategy from unconstrained movements, which can affect their duration. (2) Movements are slowed down by technological limitations of the device: deformation and migration of the fabric, friction and backlash in the Bowden cables and slack in the tendons introduce latency and affect the transient behaviour of the controller (see Additional file 1 for an estimate of the controller’s bandwidth).

The time lag, introduced by the suit, between the intention and the initiation of movement, may affect one’s feeling of being in control of his/her own actions, known as sense of agency. Previous work has shown that a longer interval between actions and their effects is associated with a lower sense of agency [60]. This idea applies to the temporal relation between motor and sensory signals too: temporally matched intended movements and proprioceptive feedback seem to be essential for promoting intuitive control and body-ownership [61]. Investigating how the kinematic imperfections of the suit impact on the user’s subjective perception of the device would be of great interest. This is especially true for clinical applications, where the strong connection between the robot and body perception, often termed “embodiment”, is a crucial factor for functional recovery [62].

There is compelling evidence that smooth movements are characteristic of efficient and well-trained motor behaviour [49] and an assistive device should not alter this. Yet we found that wearing the exosuit significantly reduced movement smoothness. Encouragingly, the difference between the mean values of the SPARC index was fairly low: −1.76±0.10 (unpowered) and −1.82±0.14 (powered), corresponding to only a 3.4*%* drop.

Unfortunately, no investigation performing such assessment on soft exosuits exists in literature, but our results echo the findings from Jarassé [63] and Pirondini [64], reporting an increase in movement jerk and number of peaks, respectively, when subjects were assisted by a rigid exoskeleton.

The deterioration in smoothness and accuracy of movement are both imperfections in the transparency of the exosuit. They suggest that care should be taken if using the device as an assessment tool, since powered movements may not reflect the characteristics of natural movements. It should be noted, however, that participants used the device for less than 10 min, in total. It would be interesting to verify if additional training results in a mitigation of these unwanted effects. Previous studies confirm that the initial disruption of natural kinematics of movement, often seen when one first wears an assistive robotic apparatus, progressively diminishes as the subject learns to use the device [65].

Finally, we should spend a word on the device’s safety. Because of the intrinsic compliance of its transmission, the suit benefits from the features of traditional series elastic actuators: its elasticity decouples the actuator’s rotor inertia from the limb, should an impact occur, and the low impedance is preserved even in case of failure. The low mass of the device, moreover, practically eliminates inadvertent damage to the environment. The admittance control adds an additional layer of safety. This is because by imposing the relation between force and velocity, unlike direct force or position control schemes, allows to constrain the power transfer between the device and its user [39]. The major limitation lies in the low efficiency of the actuation stage, caused by the high reduction ratio needed to deliver the range of forces required by the suit. This results in low backdrivability when power is off.

Although this study demonstrates encouraging results, we acknowledge that there are a number of limitations to this work. Firstly, this study involved a small and relative young cohort of participants; this reduces the strength of the statistical findings. The participants, moreover, were all males of similar height (178 ± 0.8 cm) and weight (77.8 ± 2.1 kg). This choice was forced by the size of the available prototype of the exosuit. We have no reason to believe that the results would change for a female population or for individuals of different physical structure, if they wore a suitably-sized device.

In this study, the baseline condition for comparison was an unpowered condition and not a no-suit condition. We chose this configuration to reduce the length of testing sessions and to avoid doffing and donning the suit during bouts, which could have led to increased variability in the kinematic data. The wearable component, attached to the forearm, moreover, weighs only 170 g, distributed close to the center of the elbow joint; becuase of this, we speculate that the unpowered condition would differ very little from a no-suit condition.

Another limitation of our study is that the current version of the exosuit uses a quadrature encoder, mounted on a 3D printed link, to measure the elbow angle. The linkage structure transmits no torques and bears no loads, but it only measures the true elbow angle if aligned with the biological joint. We took care of ensuring this was the case during the donning procedure but we cannot exclude that movement of the fabric may have slightly shifted its position during operation. We believe that two of the outliers in Fig. 6b may have been caused by incorrect measurement of the joint angle. We estimated that migration of one of the anchor points, along the main axis of the forearm/arm, between 3 mm–9 mm, can result in a maximum error in measuring the joint angle between 8 deg–14 deg. We did not observe systematic displacement for the range of forces used in this study. However, for the sake of robustness, it would be appropriate to replace the encoder with a more robust sensing strategy (e.g. inertial measurement units).

In order to evaluate the effects of the device solely on elbow movements, we performed the experiment in a very controlled setting. Subjects were asked to keep the arm aligned with gravity, only move the forearm and the range of motion was limited to 90 deg for safety. We have no reason to doubt that the results obtained in this study will not generalize to functional movements, but further investigations are needed to verify this hypothesis.

The present study only evaluated the performance of the exosuit when assisting its wearer in lifting a single, relatively low weight. The dynamic tasks were performed with a 1 kg mass, held in the participants’ hand, and the static task, used to assess fatigue, were performed with a load equal to 3% of the wearers’ body mass. It would be interesting to investigate the performance of the device for varying loads. We expect the limiting factor here to be comfort rather than the maximum rated torque of the actuation stage.

Last, the admittance controller used anthropometric data to estimate the assistive torque required by each participant, based on their body mass and height; because of physiological differences among subjects, a fine tuning of the parameters, performed in the familiarization phase, was required to personalize the controller. The tuning was based on qualitative feedback from the participant and was by no means optimal.

Recently published results suggest that such individualization process could be addressed systematically and automated through optimization techniques. Zhang and colleagues [66] have shown how a control paradigm that modulates the assistance characteristics, in order to minimize the metabolic cost of human walking, can accommodate the large diversity among subjects and significantly improve performance. Ding et al. [67] have shown equally encouraging results using a Bayesian optimization technique to modulate the force profile of a soft exosuit to assist hip flexion. This gives us reason to believe that identifying a suitable cost function for the device presented here and using it to optimize its control parameters, could lead to improved quality of assistance and intuitiveness of use.

## Conclusion

The advantages of a svelte and portable exosuit for the upper limbs, able to intuitively assist its wearer and reduce the effort required to move, make it a good candidate for both industrial and clinical applications. Our results showed that the device is not exactly transparent, affecting the speed and accuracy of movements, but works in parallel with the human muscles, significantly delaying the onset of fatigue.

## Additional file

Additional file 1 Collocated admittance controller. (PDF 870 kb)

## Abbreviations

ADLs: Activities of daily living
ARV: Average rectified value
DoF: Degree of freedom
EMG: Electromyography
MJT: Minimum jerk trajectory
MNF: Median frequency
MVC: Maximum voluntary contraction
nRMSE: Normalised root mean square error
RMS: Root mean square
SEM: Standard error of the mean
SPARC: Spectral arc length
