# Supplementary material for: Physiological and kinematic effects of a soft exosuit on arm movements
Source: J Neuroeng Rehabil. 2019 Feb 22;16:29. doi: 10.1186/s12984-019-0495-y (PMC6385456; doi:10.1186/s12984-019-0495-y)
Supplement: Supplementary file 1 — Collocated admittance controller. (PDF 870 kb) [file 12984_2019_495_MOESM1_ESM.pdf]

Additional Material
Collocated Admittance Controller

Control laws

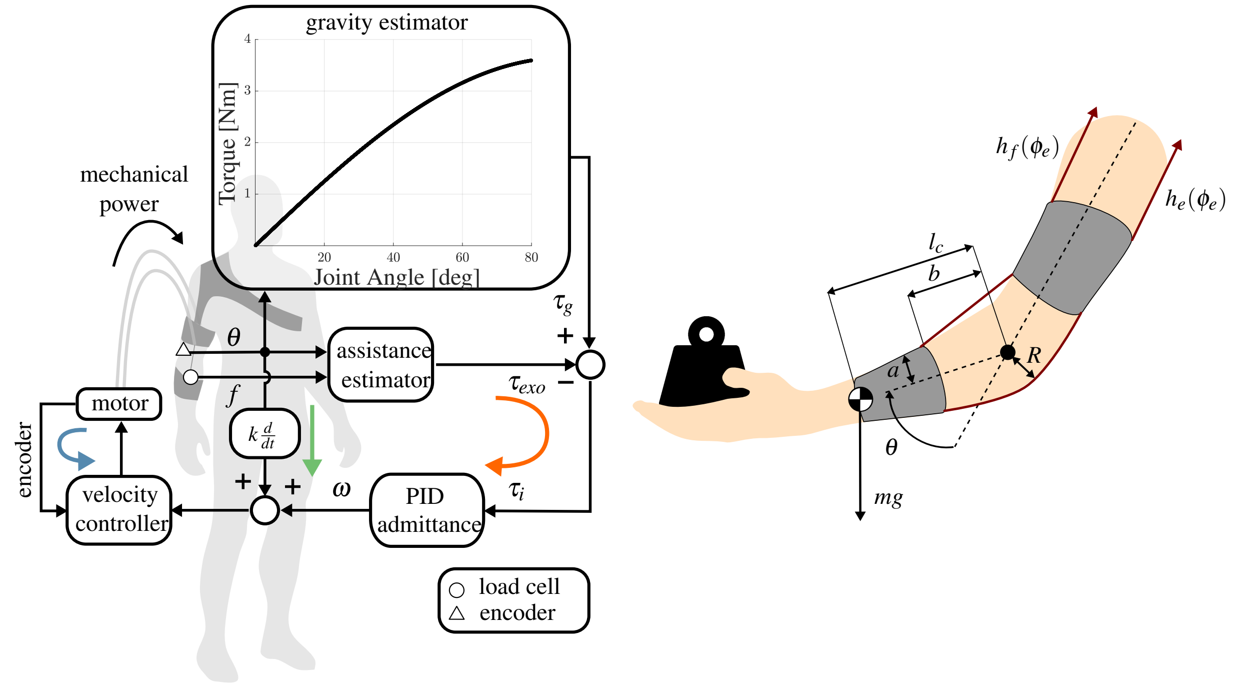

(a) (b)

Figure 1: Control schematics and tendon routing. (a) The admittance controller comprises an outer torque loop (orange) and an inner velocity loop (blue); an additional positive feedback (green) increases the suit's transparency. (b) Model of the human elbow and tendon routing. The elbow joint is modelled as a revolute joint on the sagittal plane, the geometry of the tendon routing can be used to find the extension functions and estimate the assistive torque as a function of the elbow position and cable tension.

The controller we propose for the soft exosuit for the elbow is shown in Figure 1.a. It comprises an outer torque loop and an inner velocity loop. The outer loop (indicated by the orange arrow in Figure 1.a) is responsible for tracking a position-dependent torque profile at the elbow, equal and opposite to gravity:

$$\tau_g = mgl_c \sin\theta. \tag{1}$$

The assistive torque is estimated from the tension measured by load cell on the suit's tendons. Figure 1.b shows a schematics of the suit's tendon routing. Using trigonometric relations, one can derive the mapping from a displacement of either tendon to a joint rotation; we call these extension functions, $h_f(\theta)$ for the flexor and $h_e(\theta)$ for the extensor:

$$h_f(\theta) = 2\sqrt{a^2+b^2}\cos\left(\tan^{-1}\left(\frac{a}{b}\right)+\frac{\theta}{2}\right) - 2b \tag{2}$$

$$h_e(\theta) = R\theta \tag{3}$$

where a is half of the width of the arm, b is the distance from the joint centre of rotation to the anchor points, R is the radius of the elbow joint, and θ is the joint angle. From the two extension functions $h(\theta)$ we can compute the position-dependent moment arm of the cables' tension on the elbow's axis of rotation:

$$P(\theta) = \frac{\partial h^T}{\partial \theta}(\theta) \tag{4}$$

where $h = \begin{bmatrix} h_f(\theta) & h_e(\theta) \end{bmatrix}^T$ represents the vector of cable extensions. The estimated assistive torque delivered at the joint is obtained by the equation:

$$\tau_{exo} = P(\theta) f, \tag{5}$$

where f is the measured cable tensions obtained by the load cell. This model assumes that the position of the anchor points is fixed. It neglects deformation of the fabric and soft tissues upon the application of a force from the tendons.

The difference $\tau_g - \tau_{exo}$, is converted in a velocity for the motor, ω_d, by an admittance of the form (in the Laplace domain):

$$Y(s) = \frac{\omega_d}{\tau_g - \tau_{exo}} = P + \frac{I}{s} + Ds, \tag{6}$$

with the P, I and D constants governing the characteristics of the relation between the interaction force and the motor's velocity [1].

A low-level, PI velocity loop (blue arrow in Figure 1.a) tracks the desired velocity on the motor's axis. Note that this speed loop is closed on the motor's sensor rather than on the elbow's. This approach, known as collocated admittance control [2], has been shown to robustly deal with force disturbances such as stiction and backlash, while guaranteeing stability even for low sampling rates of the high-level feedback loop [3].

Finally, a positive feedback term is added to the desired velocity (shown in Figure 1.a in green), to increase the controller's sensitivity to the user's movement: the faster the user moves, the faster the motor will. This strategy is commonly used to compensate for stick-slip friction phenomena, but it here facilitates initiation of movement, making the exosuit more transparent [4]. The final desired velocity, tracked by the low-level motor driver, has the form:

$$\omega_d(s) = P(\tau_g - \tau_{exo}) + \frac{I}{s}(\tau_g - \tau_{exo}) + Ds(\tau_g - \tau_{exo}) + ks\theta. \tag{7}$$

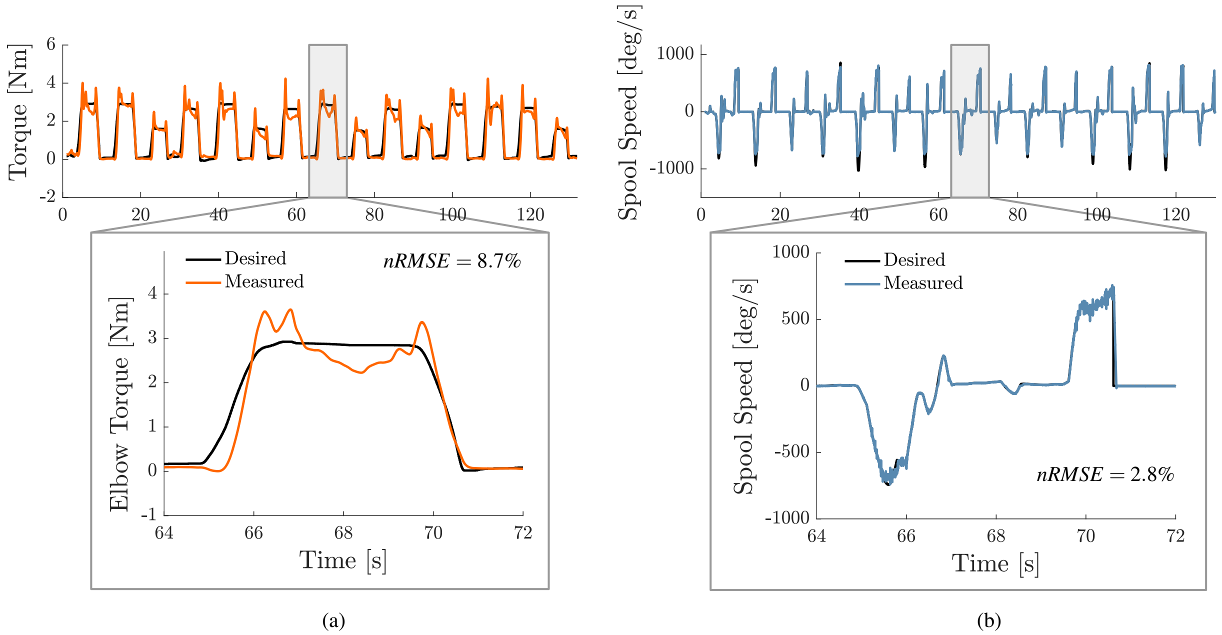

Figure 2: **Tracking accuracy of the collocated admittance controller.** (a) Accuracy of the outer torque loop in tracking a desired profile during motion of the elbow joint. The root mean square error (RMSE) is 8.7% of the range of the desired torque. (b) Accuracy of the inner velocity loop. The RMSE is 2.8% of the range of the desired velocity.

Control accuracy

The accuracy of the admittance controller in tracking the position-dependent gravity profile is fundamental for the performance of the suit.

Figure 2 shows the desired and measured profiles of both layers of the admittance controller. Figure 2.a displays the tracking accuracy of the outer torque loop (orange in Figure 1.a), for one subject and movements with a peak velocity of 84 deg/s. The reference trajectory is a position-dependent estimate of the torque acting on the elbow because of the weight of the forearm. The controller shows overshoots in the rising transient region and before the downwards motion. These are responsible for the increase in negative biological torque (Figure 7 in the manuscript). The Root Mean Square Error (RMSE), over subjects and velocities, was found to be 8.7 % of the range of desired torques.

Figure 2.b, shows the accuracy of the inner velocity loop (blue arrow in Figure 1.a), tracking the speed of the pulley on the motor's shaft, for the same repetitions. This PID was tuned to be as stiff as possible, to stably reject nonlinearities in the transmission. The RMSE, over subjects and velocities, was found to be 2.8 % of the range of desired motor speeds.

Control Bandwidth

The results presented in the manuscript show a strong dependence of the performance of the exosuit on the velocity of movement. To further quantify this effect, we recruited two subjects, not among those presented in the paper, to perform a dynamic task, using the same protocol described in the manuscript (subsection Experiment) but following trajectories in a wider range and with finer intervals of velocities . Each subject performed the task in two conditions: without receiving assistance from the device (unpowered) and with assistance (powered).

The reference trajectory consisted in a sinusoidal signal of the form

$$\theta_d(t) = A_0 + A\sin(2\pi f(t)t) \tag{8}$$

with A_0 = A = 40 deg, chosen to cover the same range of motion studied in the manuscript, and f being a step-wise varying frequency in increasing steps of 0.05 Hz, between 0.05 Hz and 0.9 Hz. These values were chosen as they correspond to movements with a peak velocity between 12.5 deg/s and 226 deg/s, equivalent to 10% to 180% of the speed of the elbow in daily tasks [5]. Each frequency value was held for 20 s; the first 5 s where discarded to allow evaluation of the response at "steady state". We collected kinematic data (AMS, AS5047P, 1000 pulses/rev) and tension on the exosuits' flexing tendon (Futek, LCM300). The control algorithm and the data acquisition were handled by a National Instrument PCI6025e acquisition board, at a 1 kHz refresh rate.

Figure 3.a shows the reference, unpowered and powered trajectories for one subject and eight different velocities of movement. In the unpowered case, the participant could easily follow the reference signal. Such was not the case for powered movements, showing a net deterioration in tracking accuracy above 75 deg/s. For each frequency/velocity of the powered case we evaluated the ratio between the Fourier coefficients of the first harmonic of the measured and desired elbow angles:

$$H(f_0) = \frac{C_1(\theta_m)}{C_1(\theta_d)}. \tag{9}$$

The magnitude and phase of $H(f_0)$ were calculated to derive a Bode plot of the human-suit's position tracking accuracy, shown in Figure 3.b. Finally, to display the effect of velocity on the level of assistance provided by the device, we estimated the biological torque exerted by the wearer in percentage of the total torque required for movement ($\tau_h/\tau_{total} \times 100$, using the procedure described in subsection Data analysis of the paper). This is shown in Figure 3.c.

Our system has a bandwidth, defined as the first frequency where the gain drops below -3 dB of its steady-state value, of 0.35 Hz, corresponding to a peak velocity of movement of 88 deg/s. This is highlighted in red in Figures 3.b, where it corresponds to an average phase of 40 deg, $\approx$0.3 s of delay. In Figure 3.c, the drop in performance is clear, with the biological torque increasing to values above 70% of the total torque for speeds above the bandwidth.

These results echo the findings outlined in the manuscript and highlight the limitations of the suit in assisting highly dynamic movement. Solutions to improve the bandwidth could include the choice of a more powerful actuation stage, design of a more efficient means of transmission (e.g. stiffer Bowden cables and more ergonomic anchor points) or the addition of a feedforward term in the controller, allowing the suit to render a higher admittance without causing instability.

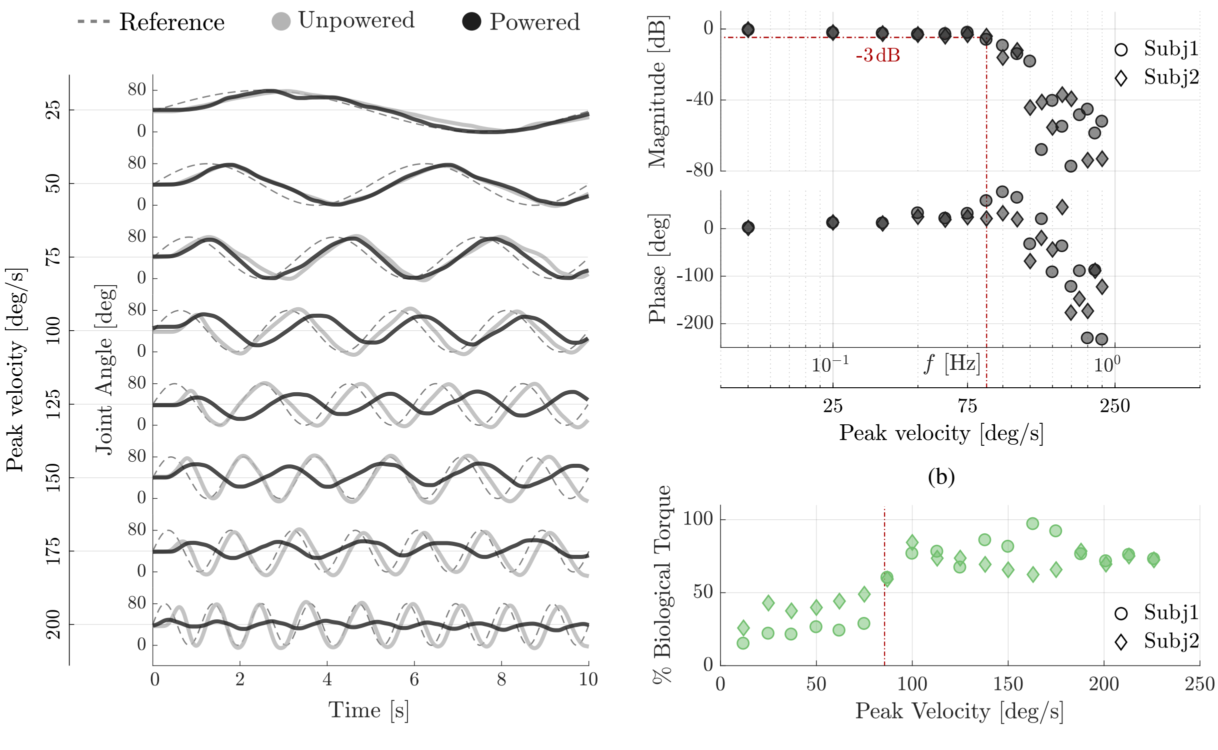

(a)

(b)

(c)

Figure 3: **Tracking and assistive performance of the suit for varying movement velocities.** (a) Reference (dashed), unpowered (gray) and powered (black) trajectories of the elbow for 8 of the 18 tested velocities of movement, shown for 10 s and one subject. (b) Bode plot of the estimated transfer function in Equation 9, between the measured and desired elbow position, in the powered condition. The human-suit system has a bandwidth of 0.35 Hz, highlighted by the red dashed line. Different markers correspond to different subjects. (c) Biological torque, in percentage of the total torque required for movement ($\tau_h/\tau_{total} \times 100$). Above the bandwidth (red dashed line), the effort that the wearer needs to exert to move increases steeply.

Bibliography

[1] Yu W, Rosen J, Li X. PID admittance control for an upper limb exoskeleton. In: Proc. 2011 Am. Control Conf.; 2011. p. 1124–1129.

[2] Calanca A, Muradore R, Fiorini P. A review of algorithms for compliant control of stiff and fixed-compliance robots. IEEE/ASME Trans Mechatronics. 2016;21(2):613–624.

[3] Pratt GA, Willisson P, Bolton C, Hofman A. Late motor processing in low-impedance robots: impedance control of series-elastic actuators. In: Proc. Am. Control Conf.. vol. 4; 2004. p. 3245–3251 vol.4.

[4] Kazerooni H, Racine JL, Huang L, Steger R. On the control of the Berkeley Lower Extermity Exoskeleton (BLEEX). In: Proc. IEEE Int. Conf. Robot. Autom.; 2005. p. 4353–4360.

[5] Buckley MA, Yardley A, Johnson GR, Cams DA. Dynamics of the upper limb during performance of the tasks of everyday living: a review of the current knowledge base. J Eng Med. 1996 jun;210(4):241–247.
